# ZEB1 Mediates Bone Marrow Mesenchymal Stem Cell Osteogenic Differentiation Partly via Wnt/β-Catenin Signaling

**DOI:** 10.3389/fmolb.2021.682728

**Published:** 2021-05-24

**Authors:** Cuidi Xu, Hongli Shi, Xin Jiang, Yongqian Fan, Donghui Huang, Xinming Qi, Qun Cheng

**Affiliations:** ^1^Department of Osteoporosis and Bone Disease, Huadong Hospital Affiliated to Fudan University, Research Section of Geriatric Metabolic Bone Disease, Shanghai Geriatric Institute, Shanghai, China; ^2^Department of Orthopedics, Huadong Hospital Affiliated to Fudan University, Shanghai, China; ^3^Center for Drug Safety Evaluation and Research, State Key Laboratory of Drug Research, Shanghai Institute of Materia Medica, Chinese Academy of Sciences, Shanghai, China

**Keywords:** ZEB1, osteogenesis, Wnt/β-catenin, postmenopausal osteoporosis, BMSCs

## Abstract

Zinc finger E-box-binding homebox 1 (ZEB1) is a zinc-finger transcription factor best known for its role in promoting the epithelial-mesenchymal transition, which is also related to osteogenesis. Here, ZEB1 was investigated for its role in the commitment of bone marrow mesenchymal stem cells (BMSCs) to osteoblasts. *In vitro*, ZEB1 expression decreased following osteogenic differentiation. Furthermore, silencing of ZEB1 in BMSCs promoted osteogenic activity and mineralization. The increase in osteogenic differentiation induced by si-ZEB1 could be partly rescued by the inhibition of Wnt/β-catenin (si-β-catenin). *In vivo*, knockdown of ZEB1 in BMSCs inhibited the rapid bone loss of ovariectomized (OVX) mice. ZEB1 expression has also been negatively associated with bone mass and bone formation in postmenopausal women. In conclusion, ZEB1 is an essential transcription factor in BMSC differentiation and may serve as a potential anabolic strategy for treating and preventing postmenopausal osteoporosis (PMOP).

## Introduction

Bone marrow mesenchymal stem cells (BMSCs) are multipotent cells that represent a promising source for regenerative medicine. BMSCs are capable of osteogenic, chondrogenic, and adipogenic differentiation (Bartscherer et al., 2006); regeneration of cells in injured tissues requires the ability to maintain differentiation toward the desired cell fate, and therefore, cellular and molecular signaling pathways and micro-environmental changes have been studied to understand the role of cytokines, chemokines, and transcription factors on the differentiation of BMSCs. The differentiation of BMSCs can be genetically manipulated and inhibited by specific transcription factors associated with a particular cell lineage.

In this study, we critically investigated the role of the transcription factor, zinc finger E-box-binding homebox 1 (ZEB1), and related signaling pathways that affect the differentiation of BMSCs toward osteoblasts. ZEB1, a member of the zinc finger E-box-binding protein family, is a transcription factor that triggers the epithelial-to-mesenchymal transition (EMT) (Bracken et al., 2008; Sanchez-Tillo et al., 2010). A previous investigation reported that ZEB1 is involved in early skeletal development. Yang et al. reported that ZEB1, which was downregulated as BMSCs were differentiated to osteoblasts, repressed the differentiation of C2C12 myoblasts into the osteoblast lineage induced by BMP_2_ (Yang et al., 2007). However, mice with a homozygous mutation in ZEB1 display skeletal defects in craniofacial bones, ribs, intervertebral disc, sternum, and limb skeletons (Takagi et al., 1998). Recently, Rong et al. (Fu et al., 2020) reported that specific deletion of ZEB1 in the endothelial cells (ECs) could reduce bone angiogenesis and osteogenesis, and injecting a pcDNA3.1 + C-eGFP-ZEB1 vector that expressed enhanced ZEB1 protein in bone ECs restored the impaired bone formation in ovariectomized (OVX) mice, suggesting that ZEB1 plays a significant role in crosstalk between angiogenesis and osteogenesis. ZEB1 was shown to be closely related to the EMT in osteosarcoma, and miR-126 and miR-708-5p played an inhibitory role in the proliferation and invasion of osteosarcoma cells by directly targeting ZEB1 and subsequently suppressed the EMT (Jiang et al., 2017; Feng et al., 2020). Therefore, the function of ZEB1 is extremely complex and may even have contradictory effects on bone metabolism, and its role in osteogenesis in osteoblast lineage deserves further investigation.

## Materials and Methods

### Cell Culture

Human bone marrow-derived mesenchymal stem cell lines were purchased from Cyagen (Shanghai, China). BMSCs were maintained in hBMSC basal medium (Cyagen). BMSCs were re-plated at a density of 8,000 cells/100 μl and were subcultured when they were 80–90% confluent.

### Osteogenic Differentiation and Alizarin Red Staining

The differentiation medium used in this study was human BMSC osteogenic differentiation basal medium purchased from Cyagen. Briefly, BMSCs were seeded at 5,000cells/100 μl in 96-well plates. For evaluating mineralization, cells were induced for 21 days, washed 1–2 times with PBS, and fixed with 4% paraformaldehyde for 30 min. This was supplemented with 50μl of freshly prepared alizarin red S solution (Cyagen), and cells were incubated for 3–5 min.

### Clinical Bone Sample Preparation

A total of 20 bone specimens were collected from patients (aged 70–85 years) who had a fragile fracture that necessitated hip arthroplasty surgery at the department of orthopedics in the Huadong Hospital. All patients were women who had incurred a low-energy hip fracture and had undergone arthroplasty within 48 h following the fracture. Individuals were excluded if they had malignant, metabolic, or endocrine diseases; chronic renal failure; or other severe systemic diseases. We collected the trabecular bone core from the intertrochanteric region of the proximal femur because it is not exactly the fracture site and is available during arthroplasty. All clinical procedures were approved by the Ethics Committee of Huadong Hospital affiliated with Fudan University (2019K055).

### Alkaline Phosphatase Activity and ALP Staining

For measuring alkaline phosphatase (ALP) activity, cells were cultured for 7 days and incubated with p-nitrophenyl phosphate (pNPP) in 1 M diethanolamine buffer containing 0.5 mM MgCl_2_ (pH 9.8) at 37°C for 15 min. The absorbance was measured at 405 nm. For ALP staining, the BCIP/NBT Alkaline Phosphatase Color Development Kit (Beyotime, Shanghai, China) was carried out according to the instructions of the manufacturer.

### RNA Interference

Negative control siRNA (si-NC) and ZEB1-specific siRNAs (si-ZEB1) were purchased from RiboBio (Guangzhou, China). BMSCs were cultured to 75–80% confluence in six-well plates and were transfected with 40 nM of ZEB1 siRNAs using RNAiMAX (Invitrogen, United States). After transfection for 24 h, the medium was replaced with an osteogenic medium to determine their cell fate-specific differentiation potential.

### Lentiviral Vector Preparation and Infection

For ZEB1 overexpression, lentiviruses expressing ZEB1 and empty vectors were purchased from OBIO (Shanghai, China). BMSCs were infected with these viruses in the presence of polybrene for 24–48 h. The packaged lentiviruses were named lenti-NC and lenti-ZEB1.

### Reverse Transcription and Real-Time PCR

Total RNA was isolated from BMSCs using TRIzol reagent (Takara, Japan). cDNA was obtained using a PrimeScript RT Master Mix (Takara, Japan) according to the instructions of the manufacturer. Relative sequences are listed in the Supplementary Materials.

### Western Blotting

Bone marrow mesenchymal stem cells were lysed in RIPA lysis buffer, and 30 ng of protein was loaded and separated by 10% SDS−polyacrylamide gel electrophoresis, followed by transfer onto membranes, which were then blocked in non-fat milk (5%) for 1–2 h at room temperature. Membranes were then incubated overnight at 4°C with primary antibodies against GAPDH (1:3,000, Proteintech, Chicago, TL, United States), β-catenin (1:1,000, Proteintech), ZEB1 (1:1,000, Proteintech), and smad3 (1:1,000, Cell Signaling Technology, Beverly, MA, United States). Membranes were washed three times with Tris-buffered saline with 0.1% Tween^®^ 20 Detergent (TBST), followed by incubation with secondary antibodies for 45–60 min.

### *In vivo* Studies

Sham and OVX-operated mice (4 weeks old, 13–15 g) were used to determine the effect of ZEB1 *in vivo*. All female C57BL/6J mice were purchased from Shanghai Model Organisms (Shanghai, China). A recombinant adeno-associated virus (AAV9) for the knockdown of ZEB1 in osteoblast lineage cells in the bone was purchased from HANBIO (Shanghai, China). Recombinant adeno-associated virus9 (rAVV9) is highly effective for transducing osteoblast lineage cells in the bone, as reported in Yang et al. (2019). A total of 24 mice were divided into four groups: (1) sham group: mice were subjected to sham operation; (2) OVX group: mice were subjected to bilateral oophorectomy to establish the osteoporosis model; (3) OVX + AAV9-amiR-Ctrl group: OVX mice were injected with empty adenovirus; and (4) OVX + AAV9-amiR-ZEB1 group: OVX mice were injected with ZEB1 knockdown adenovirus. A volume of 200 μl of rAVV9 carrying Ctrl or ZEB1 was intravenously injected through the tail at 4 weeks after model establishment. At 12 weeks after model establishment, mice in all groups were euthanized after anesthesia, and femurs were collected for μCT or bone histomorphometry. Each procedure was performed under aseptic conditions, and general anesthesia was carried out in accordance with the Animal Ethics and Experimental Safety of Fudan University (JS-004).

### Micro-Computed Tomography and Bone Histomorphometry

Femurs were fixed in 4% paraformaldehyde for 12 h and transferred to 75% ethanol at 4°C. Samples were then used for micro-computed tomography (μCT), and relative images were analyzed by SCANCO evaluation software to perform the three-dimensional structural parameter analysis including bone mineral density (BMD), bone volume/tissue volume (BV/TV), trabecular thickness (Tb.Th), and trabecular separation (Tb.Sp). For assessing new bone formation, we injected tetracycline (25 mg/kg) at 14 days before the mice were euthanized, and calcein (5 mg/kg) was injected at 7 days prior to sacrifice. The bone mineral apposition rate (MAR, μm/d) was measured by image analysis software (ImageJ; NIH, Bethesda, MD, United States). Undecalcified sections were subjected to Goldner’s trichrome staining and examined and photographed using a high-quality microscope.

### Statistical Analysis

Differences between two groups were tested by independent sample *t*-test and one-way analysis of variance (ANOVA); the Tukey’ *post hoc* test was applied to compare three or more groups. Correlation analysis was used to test the association of ZEB1 expression in bone tissue with BMD and with osteogenesis genes in bone tissue. Each experiment *in vitro* was repeated three times with similar results. All data were analyzed by GraphPad Prism 7 software and presented as means plus standard deviation. ^∗^*p* < 0.05, ^∗∗^*p* < 0.01, and ^∗∗∗^*p* < 0.001 were considered indicative of statistical significance.

## Results

### ZEB1 Expression Was Negatively Related With BMD and Osteogenesis Genes in Bone Tissue in Postmenopausal Women

**FIGURE 1:**
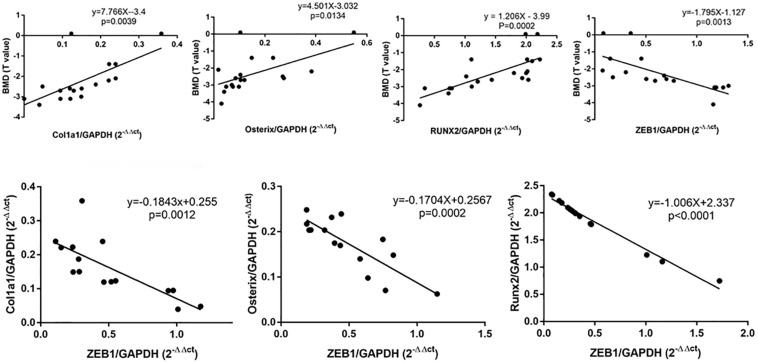
qRT-PCR analysis showed that Col1a1, osterix, and RUNX2 were significantly decreased in PMOP patients-derived bone tissue. Correlation analysis demonstrated that ZEB1 was negatively correlated with BMD and Col1a1, osterix, and RUNX2 in bone tissue derived from PMOP patients.

We detected the mRNA expression of ZEB1 in bone tissue from postmenopausal osteoporosis (PMOP) patients, and results showed that in PMOP patients, the expression of osteogenesis genes such as osterix, RUNX2, and Col1a1 in bone tissue was positively related with BMD, while ZEB1 expression was negatively related with osteogenesis genes and BMD (Figure 1).

### Downregulation of Endogenous ZEB1 Promotes Osteogenesis of hBMSCs *in vitro*

**FIGURE 2:**
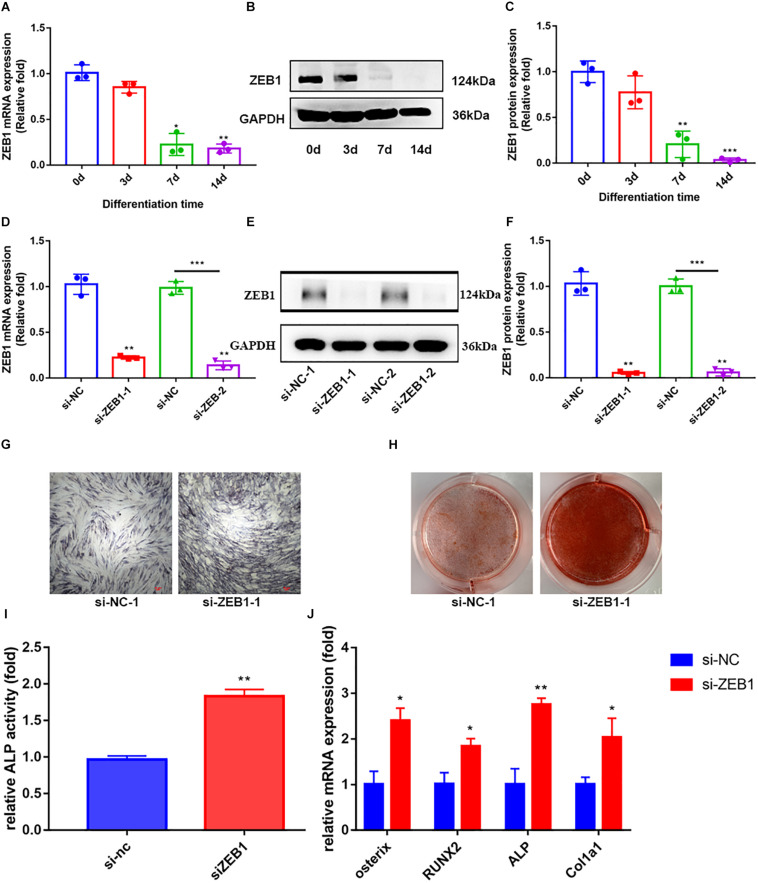
Zinc finger E-box-binding homebox 1 (ZEB1) knockdown enhanced the osteogenesis of hBMSCs. **(A)** qRT-PCR analysis demonstrated that the mRNA expression of ZEB1 decreased after osteogenic differentiation. **(B,C)** Western blotting analysis showed that the protein expression of ZEB1 decreased after osteogenic differentiation. **(D)** ZEB1 expression decreased after siZEB1 treatment – evaluated by qRT-PCR. **(E,F)** ZEB1 expression decreased after siZEB1 treatment – evaluated by western blotting. **(G,H)** ALP staining and alizarin red staining of hBMSCs after transfecting with siZEB1. **(I)** ALP activity of hBMSCs after transfection with siZEB1. **(J)** qRT-PCR analysis demonstrated that siZEB1 transfection increased the mRNA level of ALP, osterix, RUNX2, and Col1a1. *p-value < 0.05, **p-value < 0.01 and ***p-value < 0.001.

By assaying the endogenous expression of ZEB1 during hBMSCs osteogenesis, we demonstrated that ZEB1 expression in hBMSCs was significantly decreased at days 4, 7, and 14 following osteogenic differentiation at both the mRNA (Figure 2A) and protein levels (Figures 2B,C).

To explore the effect of ZEB1 on osteogenesis, siRNA-mediated knockdown was performed to downregulate ZEB1 expression, and the efficiency of knockdown was tested at 48 h post-infection by real-time PCR (Figure 2D) and western blot analysis (Figure 2E,F). ZEB1 mRNA and protein levels were reduced by over 70 and 90%, respectively, after siRNA transfection. hBMSCs transfected with si-ZEB1 showed higher ALP activity (Figure 2I) and enhanced ALP staining (Figure 2G) and alizarin red staining (Figure 2H) compared with siNC. Consistent with these changes, qRT-PCR showed that mRNA levels of ALP, osterix, RUNX2, and Col1a1 were significantly increased in the si-ZEB1 group on day 7, compared with the siNC group (Figure 2J).

### Upregulation of Endogenous ZEB1 Inhibits Osteogenic Differentiation of hBMSCs *in vitro*

**FIGURE 3:**
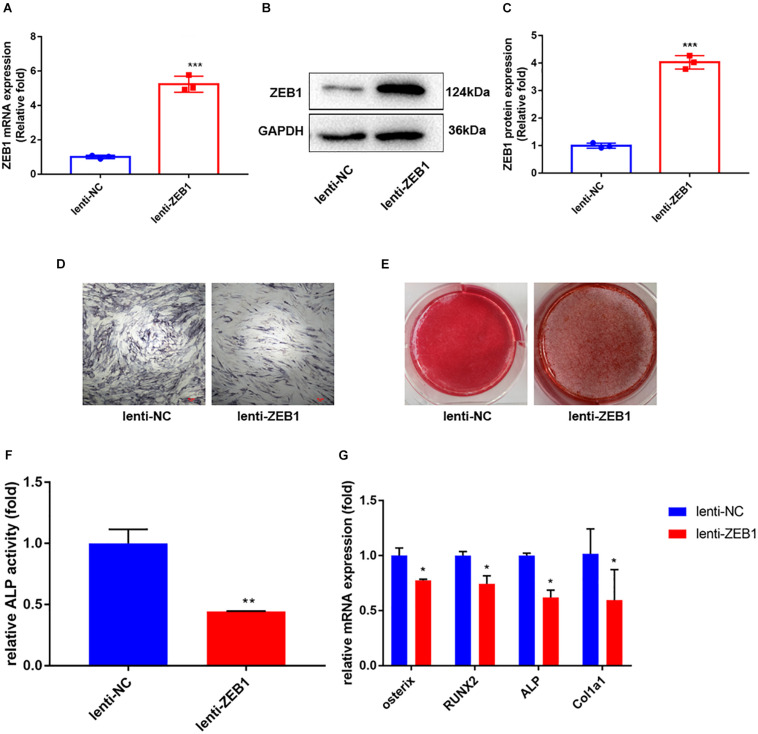
Zinc finger E-box-binding homebox 1 (ZEB1) upregulation inhibited the osteogenesis of hBMSCs. **(A)** ZEB1 expression increased after lenti-ZEB1 treatment – evaluated by qRT-PCR. **(B,C)** ZEB1 expression increased after lenti-ZEB1 treatment – evaluated by western blotting. **(D,E)** ALP staining and alizarin red staining of hBMSCs after lenti-ZEB1 treatment. **(F)** ALP activity of hBMSCs after lenti-ZEB1 treatment. **(G)** qRT-PCR analysis demonstrated that lenti-ZEB1 treatment decreased the mRNA level of ALP, osterix, RUNX2, and Col1a1. *p-value < 0.05, **p-value < 0.01 and ***p-value < 0.001.

To further investigate the role of ZEB1 in the osteogenesis of hBMSCs, lentivirus-infected hBMSCs were induced to differentiate into the osteogenic lineage. The overexpression efficiency of ZEB1 was tested after 48 h of infection by real-time PCR (Figure 3A) and western blot analysis (Figures 3B,C). Levels of mRNA and protein were increased by nearly fivefold compared to the control group.

As elucidated by ALP activity (Figure 3F) and ALP staining (Figure 3D), compared with the lenti-NC group, the expression and activity of ALP clearly decreased in lenti-ZEB1-infected cells. Alizarin red staining demonstrated that calcium mineralization was also reduced (Figure 3E). qRT-PCR showed that mRNA levels of ALP, osterix, RUNX2, and Col1a1 were significantly decreased in the overexpression lenti-ZEB1 group compared with the lenti-NC group (Figure 3G).

### si-β-Catenin Counteracted ZEB1 Downregulation-Induced Osteogenesis

**FIGURE 4:**
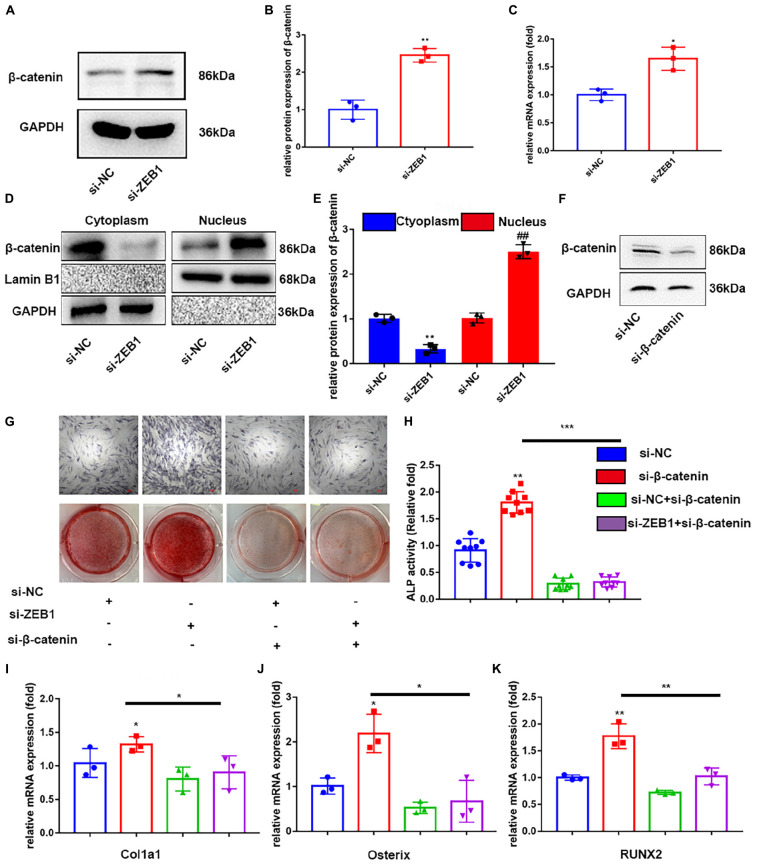
Downregulation of endogenous ZEB1 activated wnt/β-catenin signaling pathway. **(A,B)** Western blotting analysis showed that ZEB1 knockdown promoted the protein level of β-catenin. **(C)** qRT-PCR analysis revealed that ZEB1 knockdown promoted the mRNA level of β-catenin. **(D,E)** β-catenin protein level was increased in the nucleus evaluated by qRT-PCR. **(F)** β-catenin expression was decreased after siβ-catenin treatment – evaluated by western blotting. **(G)** ALP staining and alizarin red staining of hBMSCs after siβ-catenin and siZEB1 treatment. **(H)** ALP activity of hBMSCs after siβ-catenin and siZEB1 treatment. **(I–K)** qRT-PCR exhibited that siβ-catenin transfection rescued the increased ALP, osterix, RUNX2, and Col1a1 induced by siZEB1. *p-value < 0.05, **p-value < 0.01 and ***p-value < 0.001.

Previous studies have demonstrated that the differentiation of BMSC into osteoblasts is regulated by various signaling pathways, including the important Wnt/β-catenin pathway (Fan et al., 2018). To further determine if si-ZEB1 promotes osteogenesis through Wnt/β-catenin signaling, we measured mRNA (Figure 4C) and protein (Figures 4A,B) levels of β-catenin expression after si-ZEB1 treatment. The si-ZEB1 group showed significantly increased expression of β-catenin, especially in the nucleus (Figures 4D,E), along with Oct4, Cyclin D1, C-myc, and CD44 compared to the control (Supplementary Materials). However, western blotting showed that there was no significant difference in the smad3 expression between the si-ZEB1 group and the control (Supplementary Materials).

To further confirm that enhanced osteogenesis of si-ZEB1 was correlated with activated canonical Wnt signaling, the specific inhibitor, si-β-catenin, was added to the cell culture in the osteogenic induction medium. The efficacy of inhibition of Wnt/β-catenin signaling was tested by western blotting (Figure 4F), which showed that β-catenin was remarkably downregulated by this inhibitor. Importantly, with si-β-catenin treatment, the enhanced ALP activity (Figure 4H) and ALP staining (Figure 4G) in the si-ZEB1 group was abolished. Alizarin red demonstrated that fewer bone nodules were present in the si-β-catenin group compared with the si-ZEB1 group (Figure 4G), and there were decreased mRNA levels of the osteogenic genes ALP, osterix, RUNX2, and Col1a1 in the si-β-catenin group (Figures 4I–K).

### Downregulation of ZEB1 Attenuated the Bone Loss Induced by Ovariectomy

**FIGURE 5:**
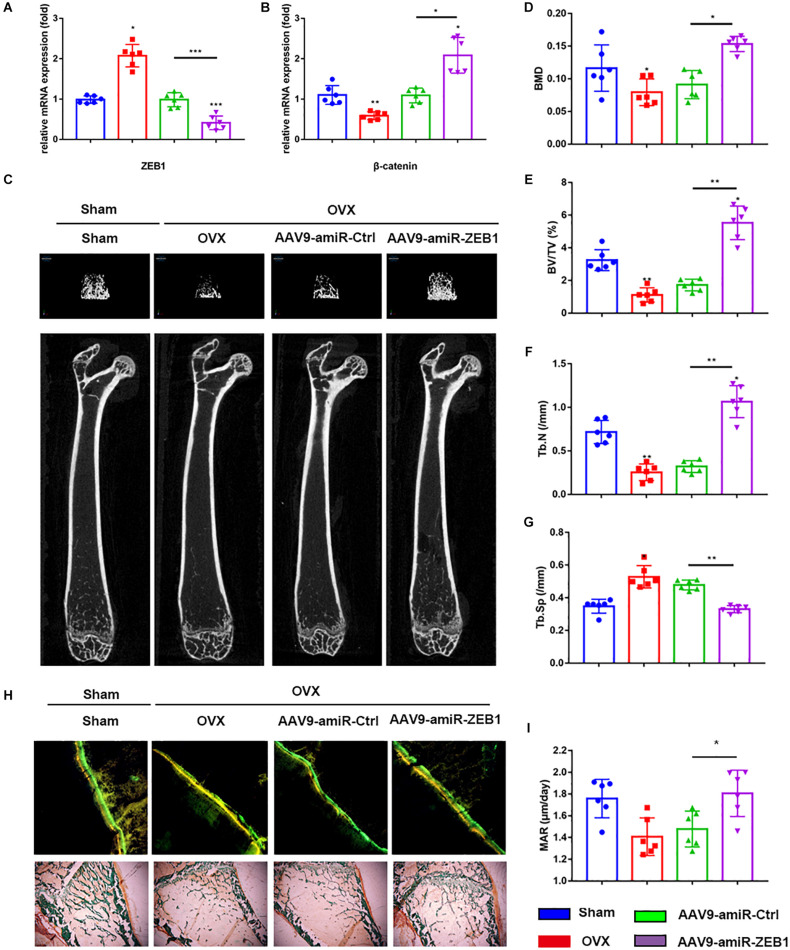
Downregulation of ZEB1 attenuates the bone loss induced by ovariectomy. **(A)** qRT-PCR analysis revealed that ZEB1 was decreased after injection with AAV9-amiR-ZEB1. **(B)** qRT-PCR analysis revealed that ZEB1 knockdown promoted the mRNA level of β-catenin *in vivo*. **(C–G)** Representative images of microCT. *n* = 6 biologically independent samples. 3D structural parameters-BMD, BV/TV, Tb.N, and Tb.Sp of femurs measured by microCT in all groups. **(H,I)** Representative images showing new bone formation were assessed by double fluorescence labeling of calcein and tetracycline and Goldner’s trichrome staining of femurs. 100×. Histomorphometric analysis of the bone formation-related parameter MAR in all groups. *p-value < 0.05, **p-value < 0.01 and ***p-value < 0.001.

To investigate the anabolic effect of ZEB1 *in vivo*, we injected AAV9-amiR-Ctrl and AAV9-amiR-ZEB1 into OVX mice. ZEB1 mRNA levels were reduced by over 60% (Figure 5A), and β-catenin increased after injection with AAV9-amiR-ZEB1 (Figure 5B). μCT and relative images were analyzed to perform the three-dimensional structural parameter analysis including BMD, BV/TV, Tb.Th, and Tb.Sp (Figures 5C–G). Compared to sham mice, OVX mice exhibited reduced bone mass, indicated by a 19.73% decrease of BMD, 58.40% decrease of BV/TV, 55.74% decrease of Tb.N, and 31.68% increase of Tb.Sp. Moreover, AAV9-amiR-ZEB1 treatment significantly increased the bone mass, demonstrated by micro-CT analysis, with an increase of BMD, BV/TV, and Tb.N and a decrease of Tb.Sp compared with both the OVX group and the AAV9-amiR-Ctrl group. Interestingly, compared with the sham group, OVX + AAV9-amiR-ZEB1 showed a 17.93% increase of BMD, 32.09% increase of BV/TV, 27.67% increase of Tb.N, and 9.28% decrease of Tb.Sp (Figures 5C–G). Similarly, the bone formation ability *in vivo* was clearly increased in the ZEB1 knockdown group, as confirmed by the Goldner’s trichrome staining and double fluorescence labeling of calcein and tetracycline, which measured the bone formation-related parameter MAR (Figures 5H–I).

## Discussion

The balance between the activities of bone-forming osteoblasts and bone-resorbing osteoclasts is fundamental to the strength and integrity of bone (Yi et al., 2017). With aging, the balance is swayed in favor of bone resorption so that the osteoclast activity exceeds the osteoblast activity, causing the bones to become brittle and prone to fracture (Ishtiaq et al., 2015). Age-related osteoporosis is related to a deficit of bone formation caused by reduced capacity in proliferation and differentiation of BMSCs (Kiernan et al., 2016). Therefore, unveiling the mechanism that regulates the differentiation of osteoblasts is of great therapeutic potential. Here, we showed that ZEB1 is endogenously expressed in BMSCs, and the expression pattern of ZEB1 during the osteogenic differentiation period also showed that ZEB1 gradually decreased over time.

The osteogenic activity of BMSCs decreases progressively as a function of increasing lifespan, and a decrease in the number of BMSCs and a reduction in their osteogenic capacity are the main causes of osteoporosis in the elderly (Qadir et al., 2020). Darryl et al. reported that ZEB1 was highly expressed in early limb mesenchyme, and ZEB1 expression was lost in these cells after condensation; by binding to a CACCT sequence contained within an E2 box, ZEB1 was shown to inhibit the activity of the rat Col2a1 promoter, suggesting that ZEB1 was involved in skeletal patterning during limb development and can serve as a negative regulator of chondrocyte-specific genes (Murray et al., 2000; Terraz et al., 2001). However, ZEB1 has not been reported to be functionally related to the osteogenesis of BMSCs. We found that silencing ZEB1 positively increased the osteogenic potential of BMSCs *in vitro*. ZEB1 silencing promoted ALP activity and mineralization and increased the expression of osteogenesis markers such as ALP, osterix, RUNX2, and Col1a1, indicating that ZEB1 is likely to inhibit the osteogenesis of BMSCs. In order to verify the possible role of endogenous ZEB1 in BMSCs, the lentivirus-based approach was applied to upregulate ZEB1 expression. Overexpression of ZEB1 limited the osteogenic differentiation ability compared to the NC group. Moreover, ZEB1 is inappropriately highly expressed in various malignant tumors and functions as a stimulating factor of tumor invasion and bone metastasis. The conditioned medium derived from ZEB1-overexpressing MDA-MB-231 cells can stimulate osteoclast maturation by upregulating MMP-1 and subsequently promote breast cancer metastasis to bone (Hu et al., 2011). ZEB1 promotes tumor invasion and metastasis by inducing the EMT in osteosarcoma, causing carcinoma cells to acquire cancer stem cell properties such as self-renewal. Our findings are consistent with the results investigated by Manuel et al., who found that MSCs from complete knockout of ZEB1 mice (ZEB1^*flox/flox*^) exhibited increased ALP staining and alizarin red staining. Manuel also translated his findings to the cancer context and demonstrated that high ZEB1 expression can prevent osteogenesis in osteosarcoma cells and eventually lock the tumor cells in an aggressive, undifferentiated state (Hu et al., 2011), indicating that ZEB1 may maintain the stemness of stem cells in both physiological and pathophysiological conditions. Therefore, in patients with decreased bone mass caused by tumor bone metastasis, targeting ZEB1 for therapeutic purposes may serve to “kill two birds with a stone.”

Activation of the Wnt/β-catenin signaling is crucial during the osteogenic differentiation of BMSCs (Gozo et al., 2013; Shen et al., 2020). The Wnt family comprises 19 closely secreted glycoproteins that mediate multiple cellular and biological processes (Cadigan and Nusse, 1997), including the formation of the body axis, development of the central nervous system, axial specification in limb development, and mouse mammary gland development. It has been reported that Wnt signaling stabilizes and causes the accumulation of β-catenin (Kikuchi, 2000; Bartscherer et al., 2006), which is an important trigger of osteoblastic differentiation. ZEB1 also has a Smad-binding domain (SBD), which binds to Smad family proteins and regulates TGF-β/BMP signaling (Postigo et al., 2003). In addition, the odontoblastic differentiation of murine dental papilla cells (mDPCs) was repressed in ZEB1-silenced cells. In the case of tooth germ at the cap stage, ZEB1 expression was lost in the epithelium-separated dental mesenchyme, but was recovered by BMP4 signaling (Xiao et al., 2021). Therefore, the function of ZEB1 in the differentiation of mDPCs was achieved via the BMP pathway. It is possible that the promoting function of ZEB1 depends on BMP/smad signaling, and its inhibitory regulation relies on Wnt signaling. In this study, we did not find any differences in Smad3 protein levels after siZEB1 treatment; however, the expression of β-catenin was significantly enhanced, suggesting that the effect of ZEB1 in osteogenesis is regulated via the Wnt/β-catenin pathway rather than the TGF-β/BMP pathway. Some studies have also reported that ZEB1 correlated with β-catenin levels. Xinghai et al. found that conditional β-catenin knockdown led to the decrease of ZEB1 expression, which, in turn, inhibited bone metastasis in lung cancer (Yang et al., 2015). Tan et al. (2019) demonstrated that the upregulation of ZEB1 induced by β-catenin could enhance the differentiation of BMSCs into hepatocytes. Moreover, hypoxia improved vasculogenesis in distraction osteogenesis through Wnt/β-catenin signaling, with a concomitant decrease in ZEB1 (He et al., 2020). The different outcomes of these studies imply that there may be synergistic or opposite effects of the identical transcription factors in different tissues or different cell lineages. However, our results proved that ZEB1 silencing led to an increase in Wnt signaling demonstrated by the increased mRNA and protein levels of β-catenin. Furthermore, si-β-catenin, used as an antagonist of Wnt/β-catenin, rescued the increase in ALP activity, mineralization, and osteogenesis gene expression induced by ZEB1 knockdown. In conclusion, ZEB1 regulates the osteogenic differentiation of hBMSCs partly via Wnt/β-catenin signaling.

Oral bisphosphonates and intravenous receptor activator of nuclear factor kappa-B ligand antibodies are the main clinical therapeutic drugs for osteoporosis (Tella and Gallagher, 2014), which both repress osteoclast activity. However, there are currently limited drugs for promoting osteogenesis. Teriparatide has been used as an anabolic drug in most countries, but it poses a risk of developing osteosarcoma. As our *in vivo* study showed, ZEB1 could be a strong potential candidate for osteoporosis therapy. AAV9-amiR-ZEB1 markedly alleviated trabecular bone loss and promoted bone formation after being injected into OVX mice. Moreover, the changes in bone-formation markers, Goldner’s trichrome staining, and double fluorescence labeling of calcein and tetracycline indicated that the anabolic effect of AAV9-amiR-ZEB1 mainly contributes to promoting bone formation. In addition, we reported that the expression levels of ZEB1 were negatively associated with bone mass and osteogenesis markers in bone tissue from PMOP patients. These results revealed that treatment aimed at the downregulation of ZEB1 in BMSCs may provide a new strategy by enhancing the osteogenic differentiation of BMSCs.

In summary, we are the first to provide experimental and clinical evidence that ZEB1 negatively regulates osteogenesis of BMSCs. ZEB1 is involved in regulating osteoblast differentiation and maintenance of bone mass. Silencing ZEB1 expression promoted osteoblast differentiation *in vitro* and slowed bone loss *in vivo*. Clinically, ZEB1 expression was negatively correlated with bone mass and osteogenesis markers in bone tissue in PMOP patients. Our data suggested that ZEB1 could be a pathogenic factor of PMOP and a potential therapeutic target for osteoporosis.

## Conclusion

In this study, ZEB1 was investigated for its correlation with bone formation genes and BMD in the bone tissue of postmenopausal women. Furthermore, it was found that ZEB1 could regulate osteogenesis through Wnt/β-catenin signaling *in vitro*, and silencing ZEB1 enhanced bone formation *in vivo*. Thus, ZEB1 could be a valuable therapeutic target for osteoporosis and other bone disorders induced by damaged osteogenesis.

## Data Availability Statement

The original contributions presented in the study are included in the article/Supplementary Material, further inquiries can be directed to the corresponding author/s.

## Ethics Statement

The studies involving human participants were reviewed and approved by Ethics Committee of the Huadong hospital. The patients/participants provided their written informed consent to participate in this study. The animal study was reviewed and approved by Animal Ethics and Experimental Safety of the Fudan University.

## Author Contributions

CX, QC, and XQ conceptualized and designed the study. QC was responsible for the administrative support. QC, DH, and YF were responsible for the provision of study materials or patients. CX and QC were responsible for the collection and assembly of data. CX was responsible for the data analysis and interpretation. All authors contributed to the manuscript writing and made the final approval of manuscript.

## Conflict of Interest

The authors declare that the research was conducted in the absence of any commercial or financial relationships that could be construed as a potential conflict of interest.

## Abbreviations

BMSCs: bone marrow mesenchymal stem cells
ZEB1: zinc finger E-box -binding homebox 1
Col1a1: collagen 1A1
RUNX2: runt-related transcription factor 2
TGFβ: transforming growth factor beta
BMP: bone morphogenetic protein
PMOP: postmenopausal osteoporosis
BMD: bone mineral density
MAR: bone mineral apposition rate
ALP: alkaline phosphatase
EC: endothelial cell
MMP-1: matrix metalloproteinase-1.
