# Supplementary material for: ZEB1 Mediates Bone Marrow Mesenchymal Stem Cell Osteogenic Differentiation Partly via Wnt/β-Catenin Signaling
Source: Front Mol Biosci. 2021 May 24;8:682728. doi: 10.3389/fmolb.2021.682728 (PMC8183571; doi:10.3389/fmolb.2021.682728)
Supplement: Supplementary file 1 [file Data_Sheet_1.PDF]

Characteristic(20)	mean±SD
Age(years)	73.05±4.91
BMI(kg/m^2)	23.16±2.25
AKP(U/L)	62.85±15.01
Ca(mmol/L)	2.11±0.09
P(mmol/L)	0.949±0.21
Femoral neck（T value）	-2.6±0.99
