# Supplementary material for: ZEB1 Mediates Bone Marrow Mesenchymal Stem Cell Osteogenic Differentiation Partly via Wnt/β-Catenin Signaling
Source: Front Mol Biosci. 2021 May 24;8:682728. doi: 10.3389/fmolb.2021.682728 (PMC8183571; doi:10.3389/fmolb.2021.682728)

ZEB1_001 GGCCTGAAATCCTCTCGAA

ZEB1_002 GAGCAAGTGTCTGAAGAAA

β-catenin_001 CGGAGGAGAUGUACAUUCAdTdT

β-catenin_002 GAUGGGAUCAAACCUGACAdTdT

β-catenin­_003 GACCCUCUCAGAACCAAAUdTdT

ALP-F CCACGTCTTCACATTTGGTG

ALP-R AGACTGCGCCTGGTAGTTGT

RUNX2-F TGTCATGGCGGGTAACGAT

RUNX2-R AAGACGGTTATGGTCAAGGTGAA

Osterix-F CCTCTGCGGGACTCAACAAC

Osterix-R AGCCCATTAGTGCTTGTAAAGG

β-catenin-F AAAGCGGCTGTTAGTCACTGG

β-catenin-R CGAGTCATTGCATACTGTCCAT

ZEB1-F GATGATGAATGCGAGTCAGATGC

ZEB1-R ACAGCAGTGTCTTGTTGTTGT

Col1a1-F GAGGGCCAAGACGAAGACATC

Col1a1-R CAGATCACGTCATCGCACAAC

GAPDH-F GGAGCGAGATCCCTCCAAAAT

GAPDH-R GGCTGTTGTCATACTTCTCATGG


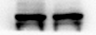
smad3


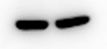
gapdh


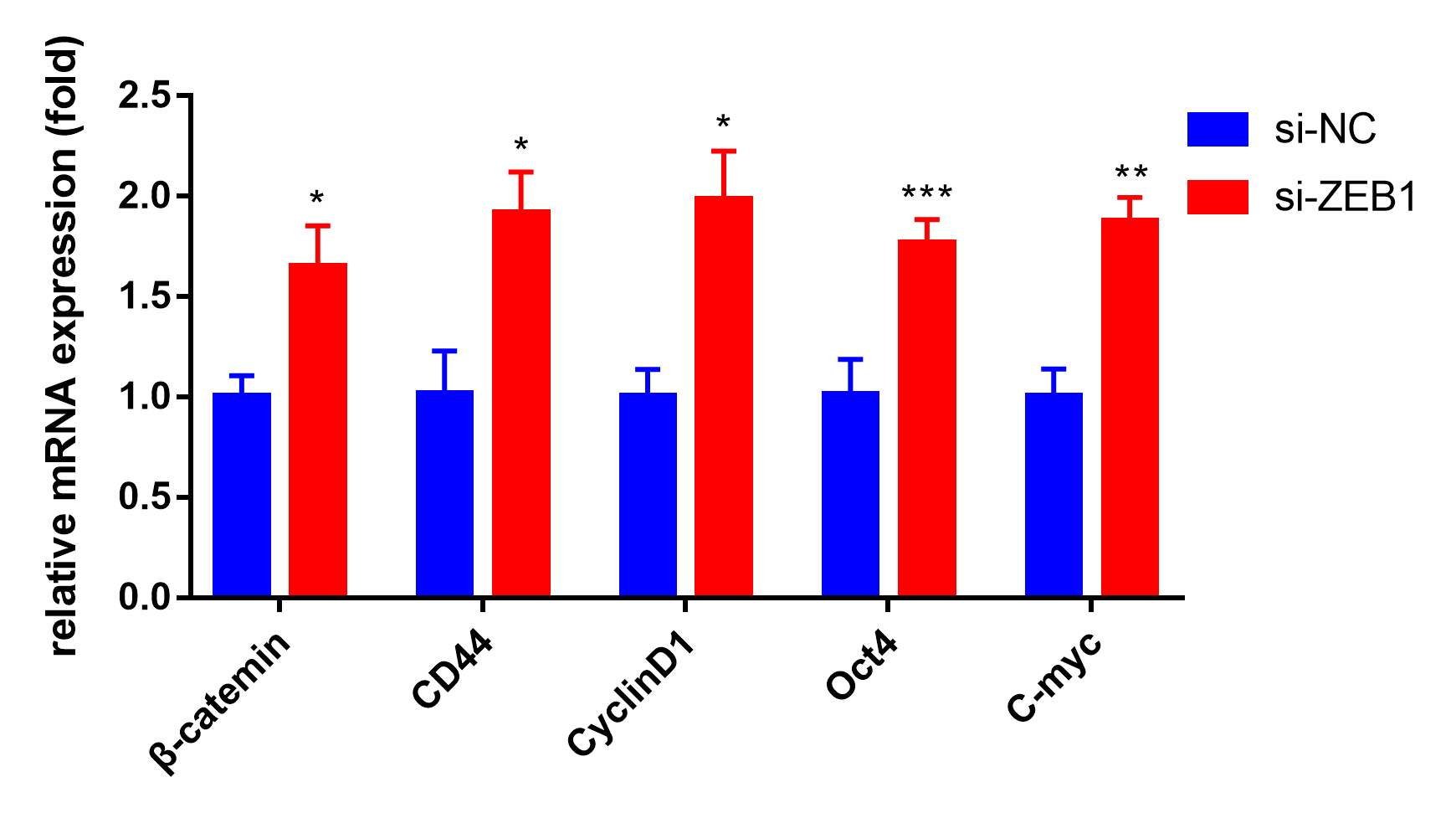

Supplement: Supplementary file 2 [file Table_1.DOCX]
